# Developing a feedback-rich culture in academic medicine: the effect of coaching and 360-feedback on physician leadership

**DOI:** 10.1186/s12909-022-03809-6

**Published:** 2022-10-24

**Authors:** Rachel Schwartz, Barbette Weimer-Elder, Elizabeth Wilkins, Dan Deka, Stephanie Wong, Bryan K. Dang, Ryan Brown, Merisa Kline, Lawrence Kwan

**Affiliations:** 1 Physician Partnership Program, Patient Experience, Stanford Health Care, 300 Pasteur Drive, MC 5603, Stanford, CA 94305 USA; 2 Foresight Collaborative, Provo, UT 84604 USA; 3 Management Department, Brigham Young University, Provo, UT 84602 USA; 4 Flying Squirrel Experiences, LLC, Boise, ID 83703 USA; 5 The Valley Foundation School of Nursing, San Jose State University, San Jose, CA 95192 USA; 6 Design Impact Engineering Program, The Department of Mechanical Engineering at Stanford University, Stanford, CA 94305 USA; 7 Division of Primary Care and Population Health, Stanford University School of Medicine, Stanford, CA 94305 USA

**Keywords:** Interprofessional team communication, Professional fulfillment, Primary care, Leadership Development; Faculty Wellness

## Abstract

**Background:**

This is a time of unprecedented change in healthcare. More physicians are being tasked with stepping into a variety of leadership roles without having received the training needed to be an effective leader. Previous data have demonstrated the effectiveness of both leadership coaching and 360-feedback tools to foster physician well-being and leadership growth. In this proof of concept study, we explore the combined effect of these two tools. The objective of this study was to examine the effect of a brief physician 360 leadership coaching intervention on perception of professional dynamics and acquired leadership skills.

**Methods:**

Participants completed a tailored 360-feedback tool to gather input on their leadership skills, then engaged in five bi-weekly leadership coaching sessions. We conducted a post-intervention semi-structured qualitative interview. Qualitative data were coded using an inductive thematic analysis approach.

**Results:**

Twenty-three primary care physicians at an academic medical center engaged in the 360 leadership coaching study. Participants reported that the intervention yielded valuable benefits in five coaching sessions. Two overarching themes emerged: a Shift in leadership awareness and Navigating their environment. Leadership awareness included increased clarity of purpose and role, and recognition that routine feedback is critical to leadership development. Navigating their environment included gaining relationship-building communication, organizational awareness and navigation strategies.

**Conclusions:**

Combining a tailored 360-feedback tool with a five-session leadership coaching intervention provided physicians with valued support infrastructure for becoming more effective leaders. Physicians described a nuanced understanding of the leadership challenges physicians face, and identified the leadership tools needed to navigate the evolving healthcare delivery landscape. Curricula for physician leadership learning could consider this combination of a customized 360 plus targeted leadership coaching for training physician leaders.

**Supplementary Information:**

The online version contains supplementary material available at 10.1186/s12909-022-03809-6.

## Background

A comprehensive synthesis of leadership studies defined a leader as someone who “selects, equips, trains, and influences one or more follower(s) who have diverse gifts, abilities, and skills and focuses the follower(s) to the organization’s mission and objectives …” [1] By this definition, all physicians are leaders. Many lead, even without managerial titles, in their daily clinical roles by guiding and navigating teams and patients through complex health decisions and scenarios. The COVID-19 pandemic’s effect on the delivery system and the way we care for patients has only increased the need for physicians to lead. While many organizations and authors have called out the current gap in leadership training for physicians and the need to develop routine leadership learning opportunities for physicians, [2–4] there is not yet consensus on the most effective tools and a ‘core’ curriculum for such programs.

Frich et al. in 2014 provided a systematic review of medical literature on physician leadership development programs to characterize the setting, educational content, teaching methods, and learning outcomes achieved [4]. The review highlighted several gaps including “...a limited use of more advanced training tools such as interactive learning and feedback in order to develop greater self-awareness” (p.656) [4]. Articles from the business world have described the pressing need to build curricula and tools that help today’s physicians build ‘systems’ and ‘interpersonal’ literacy [2].

Studies have highlighted the effectiveness of coaching in alleviating burnout and improving well-being amongst primary care physicians [5]. A 2020 systematic review of leadership development initiatives for physicians noted the value of multisource feedback and coaching as effective means of developing physician leadership capacity [6]. Studies have reported that the use of multisource feedback (in the form of a 360 tool) improves physician professionalism, interpersonal communication, teamwork, and leadership behaviors [7]. While 360 evaluations are routinely used for physician performance assessment, their use purely as a tool for professional development is relatively rare. In this proof of concept study, we employed two common leadership training tools in combination: 360 multisource feedback (using a non-proprietary 360 feedback tool) and coaching. These tools are traditionally lengthy and expensive endeavors, reserved for those in higher leadership positions or as part of an exclusive leadership course. We provided a brief and comprehensive implementation of these tools aimed at a broad cross-section of a primary care division which included many early and mid-career leaders. Our objective was to evaluate how the combined effect of these tools influenced primary care physicians’ perception of professional fulfillment, professional dynamics, and their perspectives on leadership.

## Methods

### Participant selection

This study was approved by the Stanford Institutional Review Board. Participants were recruited from physicians in the Division of Primary Care and Population Health through a nomination process in which leaders and members of the division were asked to identify candidates (including themselves) who they felt would benefit from further leadership training. Fifty physician leaders were initially invited in August 2019. Nominees were re-contacted in November 2020 with the goals and details of the program. Those who agreed to take part in the study completed an online pre-survey and selected raters to complete a customized online 360 leadership evaluation.

### 360-Feedback tool

Prior to the coaching intervention, we conducted focus groups with sixteen multidisciplinary physician leaders, asking them to rank 16 leadership competencies for physicians in order of importance (drawn from Dye & Garman [8]). After ranking these items, focus group participants were asked “What made the top 5 most compelling to you?”, “What made the bottom 5 least compelling to you?” “How does your ranking connect to your understanding of your role?” Based on their responses, we customized the leadership 360 evaluation (see Additional file 1: Appendix A for details).

To set expectations for successful participation, the study team created an informational video describing the goal of the program, the value of feedback to participants, and what the leadership coaching process involved. The video was sent to all participants and their selected raters.

The 360-evaluation tool was administered by a HIPAA-approved third-party. Participants received an email summary report 48 hours before their first scheduled coaching session.

### Coaching process

Based on previous coaching work (presented in a parallel manuscript) demonstrating changes in physician behavior as a result of coaching, as reflected by changes in patient ratings on Press-Ganey surveys, occurred at or after 3 sessions. As a result, we selected a protocol securely in that changed-behavior range, choosing 5 1:1 coaching sessions, scheduled at two-week intervals. Accommodations were made for adjustments in scheduling as needed by participants; 22 participants completed the coaching between March 22 to June 9, 2021. The remaining participant had repeated scheduling conflicts and completed the coaching on July 19, 2021. Physicians completed these sessions in addition to their existing responsibilities.

All three International Coaching Federation-certified coaches were non-physicians with strong backgrounds in education, selected for their experience with leadership development and 360 evaluations; one had a clinical nursing and military background followed by a PhD in Leadership (BWE), one had a PhD in Instructional Psychology with a focus on transformative learning and serves as a management professor at a university (EW), and one had an MBA with 20 years of leadership development and organizational change experience (DD).

To limit the effect of variable coaching styles and techniques, coaches engaged in 3 months of collaborative, weekly meetings to outline each element of the coaching process to ensure alignment on philosophies and processes. The resulting coaching guidelines for each session (see Additional file 2: Appendix B) served to increase standardization across participants. Coaches started the initial coaching session by asking about the participant’s previous experience with 360 evaluations, then clarified how this one was unique---specifically tailored for growth rather than performance evaluation. They highlighted the confidentiality of this 360, noting that only the participant and coach were able to see the 360. The 360 results provided a springboard from which participants selected a target leadership goal to focus on in their coaching sessions.

### Interview and coding analysis process

Following the coaching intervention, participants were asked to take part in a recorded qualitative interview. The interview, conducted over Zoom, followed a semi-structured interview guide (see Additional file 3: Appendix C). The interview guide was developed collectively by the leadership coaches, a physician leader (LK), and a qualitative health services researcher (RS). It focused on participants’ perceptions of the strength and limitations of the program, its perceived impact on their leadership growth, and their ideal next steps for improving the program. Based on initial input from two pilot interviews conducted with non-participating physicians who had recently completed a similar course of leadership coaching, the interview guide was revised prior to data collection. All interviews were conducted by a researcher (RS) who was not involved in the coaching process. Participants were informed that the qualitative data would be kept separate from the coaching team in order to elicit as much candid feedback on the intervention as possible. The interviews were transcribed and only the coding team had access to the transcripts. The interviews ranged in length from 18 to 53 minutes.

The interviewer was known to three of the participants and had a collegial, non-hierarchical relationship with all. The research team discussed the value of having these three interviewed by someone unknown to the participants, but decided consistency in the interview process outweighed having an unknown interviewer. One participant (not known to the interviewer) requested that they not be audio recorded; for this participant, the interviewer took detailed notes and no direct quotes were used. The rest of the recordings were transcribed, imported into Dedoose software (Version 9.0.17 [9]), and coded by a coding team of five people: a female communication scientist/health services researcher (RS) who conducted the interviews, a male adult education and communication media technology specialist (RB), a female health services/relationship-centered communication project manager (SW), a male primary care physician and medical director (LK) and a male nurse and ed-tech startup founder (BD).

The research team maintained reflexivity by continually discussing and challenging assumptions throughout the analysis process. The analysis was additionally staged so that the physician joined the discussion only after the rest of the coding team had prepared an initial synthesis. This was designed to ensure that team members with lower authority felt comfortable presenting, and openly debating, themes. Motives between research team members varied; while three of the coders were involved in the study design and administration, each brought a slightly different lens: one focuses on physician well-being research (RS), another on project administration (SW), and the third on physician leadership development (LK). The remaining coders (RB, BD) were naïve to the study goals and their insights were shaped by their professional experience in educational design (RB), educational technology and nursing (BD). All career stages were represented on the coding team. Coaches were completely excluded from the qualitative analysis in order to avoid any potential bias.

Following an inductive thematic analysis approach, [10] and a constructionist paradigm, the coding team met weekly over a period of 2 months to develop a codebook, code the transcripts, iteratively refine the codebook and align on the most salient themes. A minimum of two coders reviewed and coded each transcript. All discrepancies were resolved through consensus.

## Results

### Participant demographics

Twenty-nine physicians enrolled and 6 chose to discontinue before the coaching began; one person declined because they were concerned about psychological safety/confidentiality, and five had scheduling conflicts. Twenty-three participated. See Table 1 for demographics.

**Table 1:** Participant Demographics

	Number of Participants, out of *n* = 23 (%)
Gender (Female)	19 (83%)
Years at Current Institution	
1–10	7 (30%)
11–20	9 (39%)
21–30	5 (22%)
31+	2 (9%)
Racial Demographics	
Asian	13 (52%)
Latinx/Hispanic	1 (4%)
Caucasian	11 (44%)

#### Qualitative themes

**Fig. 1:**
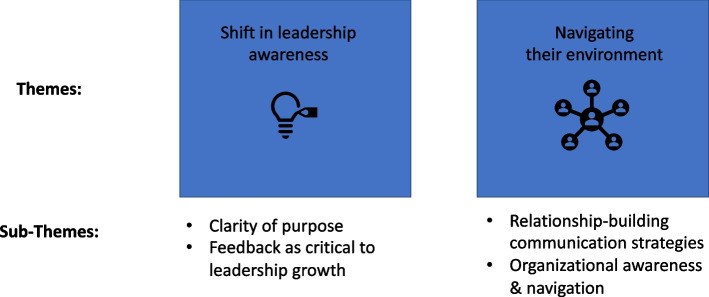
Overview of qualitative themes

Two overarching themes, Shift in leadership awareness and Navigating their environment, emerged as a result of the 360 leadership coaching intervention. These themes, and associated sub-themes, are detailed below (Fig. 1).

##### Theme 1: shift in leadership awareness

The 360-leadership coaching study affected participants’ notion of what it means to be a leader and provided insights into how their behavior was perceived by others. They described how the 360-feedback tool, the opportunity to debrief it with a leadership coach, and the dedicated time for self-reflection, were all instrumental for new insights and leadership growth.

Many participants reported hesitating to enroll in the study due to lacking an administrative title. They described how the coaching process made them reconceptualize their notion of what leadership is and reported that this shift altered their perception of team roles and responsibility towards colleagues. As one explained:

If they’re followers, I haven’t really paid that much attention to what their career growth necessarily is. I think this [program] has helped me understand that it’s just as important for us to pay attention to that, partly because it’s your responsibility. It’s part of your growth as a leader: pay attention to everybody who is following behind you.

Others explained how the program helped them recognize that leadership was not tied to an ideal performance or context but was a stance and mindset:

It’s not about always performing at the level that you want, but about when you fail or don’t live up to the expectations, about learning from that and bouncing back more quickly rather than it derailing you. I think one of the key takeaways too was just viewing oneself as a leader in all contexts, not just the certain specific title role.

Many participants noted a tendency to be overly self-critical, and described how important it was to have the coach identify their areas of competence and strength as a foundation from which to build additional leadership skills. For many, the 360-feedback provided new insights about how their behavior was affecting colleagues. As one summarized:

One of [the things I learned from the 360 evaluation] was the disconnect. I see myself much harder than other people. Then also...being able to see things from other people’s point of view. I’m too passionate and basically, need to stop yelling at people! I’m like, ‘Oh my God, I’m trying to see,’ but being able to maybe see it from their perspective a little more.

#### 1a. Sub-theme: clarity of purpose and role

The increased self-awareness for many directly contributed to improved clarity of purpose and professional role, helping them reconnect and rekindle their career goals. For some, this required setting boundaries and declining certain opportunities to better align their activities with their purpose:

Some of the things I was doing, honestly, just didn’t really feel authentic for me in terms of what I enjoy doing and what I feel good doing. I was just doing them for the sake of doing them, and because somebody higher above, somebody thought it would be good for me … It’s getting easier, but it still is hard to say ‘no’ to things, especially when someone’s like, ‘Oh, I think you’d be really good at this,’ even though it may not match with what you want to do. Especially if it’s coming from someone who you really respect and you feel like saying ‘no’ was disappointing them somehow. Which I’ve learned it’s not.

For others, the self-reflection required by the coaching process resulted in a commitment to set aside more regular time for reflection and long-range career planning:

For me personally, I feel like my growth and development at this time really comes from sitting and reflecting on what I want, what my values are, what it is to be my authentic self and bring my authentic self into the work that I do. That will ultimately give me the most satisfying career where I’m able to be the most useful to the people around me too.

Another similarly summarized:

I think it gave me a chance to refocus and to look at my current self and the future self I hope to be and a roadmap to get there.

#### 1b. Sub-theme: feedback as critical for leadership growth

Participants described how the chance to receive candid feedback about their leadership behaviors highlighted its current absence in the culture of medicine and the need for routine, ongoing feedback to support continued leadership growth. They articulated that it is incumbent on the physician to actively solicit this feedback:

I think that physicians, oftentimes we don’t get a lot of feedback like this. Oftentimes in our training, you get feedback about how well you take a history, present a patient, whether or not you got the right diagnosis. You get feedback like that, but you really do not get a lot of feedback about how you are perceived by your peers. You don’t get a lot of feedback about why you were not chosen for something. You don’t get an audit of feedback about how you can improve so that you could be an asset to your division. You just don’t get that. I think part of it is we don’t ask often, we don’t ask enough. It’s not necessarily a culture where that is common.

Part of the coaching intervention involved encouraging participants to solicit feedback from various stakeholders. Many participants described the initial discomfort involved in this process of asking for feedback and explained their epiphany that the act of asking for feedback strengthened their relationship with stakeholders:

It was very uncomfortable. It definitely pushed me out of my comfort zone. I don’t like talking about myself and … to have to ask for feedback is sometimes a little difficult. It was, yes, it was not something that I would have done [on my own], but it was good to have done. I think now the relationship is maybe is a little bit different with these various people, and so there’s some accountability.

##### Theme 2: navigating their environment

The coaching part of the 360-leadership coaching intervention resulted in participants describing having gained a set of tools for enhancing their leadership interactions. These ranged from communication strategies to new insights for how to more effectively navigate the organization.

#### 2a: sub-theme: relationship-building communication strategies

Participants explained that the coaching intervention placed an emphasis on fostering relationships, and provided the physicians with communication strategies for feeling more in control of their role in the interaction. These included concrete tools, like the two participants below describe:

I liked some of the words that [my coach] gave me that I could use so that not only would it help me with my scheduling, but it would build up the leaders below me, and then setting agendas for meetings.

We talked a lot about-- How did [the coach] put it? The shared pool of meaning in communication and how you can break down defensiveness so that I can be heard and make the other person feel heard while disagreeing or putting a new concept out there.

#### 2b: sub-theme: organizational awareness and navigation

Participants noted the division’s investment in leadership coaching for physicians changed their perception of the organization. They expressed surprise and gratitude for the organizational commitment to their individual growth as a leader, and explained how it changed their notion of their place within the community:

It made me appreciate the fact that I had that support there. I think the idea that [the division] supported this for a lot of people was pretty amazing. I think it’s certainly appreciation and gratitude for the fact that I’m in a place where people support these things. [The program] helped me remember that.

Multiple participants described how they believed this division-level investment would have a ripple effect felt throughout the organization. They asked that the coaching program be expanded to allow all colleagues to participate, and remarked on the positive return on investment:

I appreciate the organization’s commitment to me. I feel like they’re seeing me, at least some part of the people in the ether are seeing me as a person that has emotional and leadership needs in addition to just being a doctor. I’m more than just patient care. When I’m invested in, it comes out into my patients, it comes out into my coworkers. They should know that. This is good happiness retaining doctor stuff that they need to do because we have so many demands on us and so many things that drain us. To give us an opportunity to put a little bit back in, it goes a long way.

Many described how the coaches provided insight about organizational structure that allowed the physicians to feel more capable of navigating the system effectively:

One of the interesting first visualizations that [my coach] walked me through was, “Okay, if you think about [your previous institution] as a very mature organization, you need to think about [the current institution] more as a startup, that the leadership skills that worked for you at the [previous institution] can work here, but the environment is very different.” … That was like one example of just-in-time visualization and reframing that was able to help me move forward and get out of some anxiety that I was having that I’m not being effective.

They described how this increased awareness led to greater agency through having the ability to make more conscious choices, as the two participants below describe:

One thing I’ve learned is that when your style of leadership is not maybe directly aligned with or in alignment with the style of leadership of your bosses, then you have to either adapt or pivot or find new bosses, basically.

[The coach] also said that the only thing you can change is yourself, I can’t change the organization and that’s sometimes good to hear from someone else.

## Discussion

In this proof of concept study, we explored the effect of a 360-feedback tool combined with five sessions of 1:1 leadership coaching on primary care physicians’ perception of leadership skills. Using qualitative investigation, we found participants reported a shift in their perception of what it meant to be a leader and in the relational skills they had acquired over the course of the coaching program. Participants described how the 360-feedback tool provided them with a heightened self-awareness of their own leadership competencies and how their actions were perceived by colleagues; when combined with tailored leadership coaching, the physicians were able to gain concrete leadership tools that allowed them to have more agency in navigating their professional environment.

Our results echo the leadership gains found in previous physician leadership coaching studies, [3, 5, 11, 12] including reports of internal shifts in leadership perception and new approaches to conceptualizing their work and professional role [13]. However, unique features of our study included partnering with physicians to develop a tailored leadership 360-feedback tool, and a shorter duration, five-session coaching intervention. Previous studies using 360-evaluations for physician leadership purposes have similarly noted the need to pair a 360 with opportunity for coaching debriefs to optimize behavioral change [7]. Our results underscore these findings and suggest that pairing 360-feedback with a coaching intervention is effective in yielding leadership insights and providing physicians with concrete tools for fostering professional relationships.

An unexpected finding from our study was participants’ emphasis on how the leadership coaching intervention made them feel invested in and appreciated by the organization. Physicians’ perceived appreciation/feeling valued has been linked to lower odds of burnout [14]. A recent study identified chair support and compensation as two key areas of perceived appreciation [15]; our qualitative data indicate that our intervention effectively communicated division-level investment.

A key insight from the study was participants’ appreciation for the importance of routine feedback for leadership growth. They noted the current lack of a feedback-rich culture in medicine, and highlighted the historical trepidation in actively soliciting feedback from stakeholders. Interestingly, our study reflected a hunger for it, and the feedback was embraced. While there are many recent studies calling for the use of coaching with medical trainees in order to create a feedback-rich culture, [16–18] this is not yet standard practice for trainees, much less for faculty. Future investment in physician leadership skills through routine provision of leadership coaching could be instrumental in creating a feedback-rich culture in academic medicine.

### Limitations

There are a number of limitations to our study. First, this intervention occurred during the COVID-19 pandemic. This transitional, disruptive time may have allowed for greater openness to support interventions. It is possible that the qualitative data did not display the full value of the program as topics discussed with coaches required a high degree of confidentiality and psychological safety that participants did not always feel comfortable sharing with a researcher who was documenting the conversation. The study results may also have been limited by the time interval for the qualitative interviews; multiple participants mentioned that they felt unable to articulate the full impact of the program as they were being interviewed so soon after the final coaching session. Interviewing participants at longer time intervals post-intervention could provide a clearer picture of how they were able to integrate insights from the program into their professional life. Furthermore, thematic saturation was not reached due to the limited number of study participants.

As noted by McGonagle and colleagues in their coaching study, [5] despite our standardization approaches, there was some variation between coaches in their coaching style and leadership tools offered.

Finally, our study design involved an intentional selection bias: our messaging was firm about the time investment needed for physicians to participate, resulting in highly engaged physicians. Future studies could usefully explore whether this type of selection bias is essential for the success of this type of intervention or whether it is possible to achieve similar gains with a standard-issue 360 leadership coaching program. Future studies that employ mixed-methods analyses and include a control group will allow for a clearer understanding of the intervention effects.

### Future directions

Next steps include exploring how to optimize each element of the 360 leadership coaching intervention. This includes exploring the optimal number of coaching sessions to support targeted leadership growth, how frequently participants should receive repeated 360-feedback, and how best to integrate 360 leadership coaching into a larger organizational strategy for physician leadership development. Additional investigation of these factors will allow us to provide evidence-based guidance for the organization-level infrastructure needed to support physician leadership development.

It is currently unknown how this intervention affects organization-level outcome measures. We are interested in examining the effect of a 360 leadership coaching intervention on value-based care, patient experience scores, managerial competencies, and additional quality metrics that we anticipate would improve as a result of the institutional investment in leadership coaching. Additionally, future studies would usefully investigate the duration of the effect using longitudinal investigation.

## Conclusions

Leadership is a complex activity that incorporates more than just a set of concrete skills or an understanding of how to properly execute plans [4, 19]. Leadership is also a dynamic, relational activity that relies on the internal compass of the individual leader as well as their courage and wisdom to appropriately navigate unpredictable and uncharted situations [19, 20]. The ‘curriculum’ to prepare physicians for these leadership challenges and situations requires more than didactics and a checklist of competencies. Providing advanced tools like coaching and 360-feedback creates space for physicians to hone the most important tool in their leadership arsenal, themselves. Our study shows that creating this reflective space and applying these tools properly has a significant impact on their perceptions of their leadership capability and their desire to grow.

## Supplementary Information

**Additional file 1:** Appendix A.

**Additional file 2:** Appendix B.

**Additional file 3:** Appendix C.

## Data Availability

The datasets analysed during the current study are not publicly available (and were only available to a sub-set of the research team to ensure participant confidentiality and to prevent any potential analytic bias associated with the coaching relationship). Deidentified qualitative data according to theme can be made available upon reasonable request from the corresponding author.
