# Supplementary material for: Developing a feedback-rich culture in academic medicine: the effect of coaching and 360-feedback on physician leadership
Source: BMC Med Educ. 2022 Oct 24;22:733. doi: 10.1186/s12909-022-03809-6 (PMC9590387; doi:10.1186/s12909-022-03809-6)
Supplement: Supplementary file 1 — Additional file 1: Appendix A. [file 12909_2022_3809_MOESM1_ESM.docx]

Appendix A

360 Leadership Survey Procedure and Content

As one of the first steps in the 360 evaluation, faculty member participants were asked to thoughtfully select individuals (feedback providers) who would provide 360 feedback on their leadership behaviors and insights on their previous work/relationships throughout their career. Faculty were required to select a minimum of 3 and maximum of 5 individuals in each of the following categories:

- Category 1 (Leaders): Those who you perceive have hierarchical authority/ or power.
- Category 2 (Peers): Those who you perceive are colleagues and lateral to you.
- Category 3 (Followers): Those who perceive you as authority/ or power.
- Category 4 (Others): Those who have other roles (including non-clinical administrative team members) that may not fit into the other categories.

The feedback providers received an invitation to complete the 360 evaluation on behalf of the faculty member via email. Each invitation was set up and automated from the Qualtrics 360 program and included a unique link to the faculty member’s 360. Faculty providers received 3 weeks to complete the 5-10 minute survey. In addition to the invitation email, 2 additional emails including a reminder and final call were sent out. Tracking of completions was available in Qualtrics. The 360 covered the following leadership content areas:

4 Cornerstones of Exceptional Leadership Mapped to 16 Critical Competencies for Healthcare

(Dye & Garman, p.230)

| *Cornerstone* | *Domain* | *Sample question* | *Likert Rating scale* |
| --- | --- | --- | --- |
| **Well Cultivated Self-Awareness** | Leading with Conviction | Not afraid to take unpopular stance if necessary | Very poor/Poor/Fair/Average/Good/Excellent/Outstanding/Don’t know |
|  | Using Emotional Intelligence |  |  |
|  | Earning Trust and Loyalty (Result of the Action) |  |  |
| **A Real Way with People** | Listening like you mean it | Supports staff in developing their skills | Very poor/Poor/Fair/Average/Good/Excellent/Outstanding/Don’t know |
|  | Giving Great Feedback |  |  |
|  | Mentoring |  |  |
|  | Developing High Performing Teams |  |  |
|  | Energizing Staff |  |  |
| **Masterful Execution** | Mindful Decision Making | Is aware of how strategies play out in the field | Very poor/Poor/Fair/Average/Good/Excellent/Outstanding/Don’t know |
|  | Building True Consensus |  |  |
|  | Generating Informal Power |  |  |
|  | Driving Results |  |  |
|  | Stimulating Creativity |  |  |
|  | Cultivating Adaptability |  |  |
| **Compelling Vision** | Communicating Vision | Builds strong rapport over time | Very poor/Poor/Fair/Average/Good/Excellent/Outstanding/Don’t know |
|  | Developing Vision |  |  |

Open-ended questions:

1. Describe this person's greatest strengths as a leader.
2. Describe specific things this person could do to become a more effective leader.
