# Supplementary material for: Developing a feedback-rich culture in academic medicine: the effect of coaching and 360-feedback on physician leadership
Source: BMC Med Educ. 2022 Oct 24;22:733. doi: 10.1186/s12909-022-03809-6 (PMC9590387; doi:10.1186/s12909-022-03809-6)
Supplement: Supplementary file 2 — Additional file 2: Appendix B. [file 12909_2022_3809_MOESM2_ESM.docx]

Appendix B: Coaching Protocol

Session 1

Purpose

• Get to know each other, histories, expectations and coaching overview

Desired Outcomes

• Overview of coaching process

• Answer any questions they have

• Coaching initial assessment

Guiding Questions

• How did you get into medicine?

• What do you love about your job?

• What is your vision of yourself as a leader?

Session 2

Purpose

• Debrief the 360 feedback and identify initial developmental goal

Desired outcomes

• 360 debrief completed

• Development goal draft

Guiding Questions

• What strengths stood out to you in your 360?

• What surprised you in your 360?

• Which opportunity areas do you feel need to be shored up in order to reach your leadership goals?

Session 3

Purpose

• Solidify developmental goal and discuss next steps

Desired outcomes

• Finalized development goal

• Awareness of ways to make progress toward the goal

Guiding Questions

• As you reflected on potential goals, what stood out to you as the behavior that would best help you take your leadership to the next level?

• Who would have an important perspective on your behavior in this area?

• How frequently do these people see this behavior?

Session 4

Purpose

• Forward progress on participant’s behavioral goal

Desired outcomes

• Provide accountability for progress toward the goal

• Reflection on participant’s learning

Guiding Questions

• What progress did you make toward your behavioral goal?

• What did you learn from your experiments with this behavior?

• What will you do differently in the next two weeks?

Session 5

Purpose

• Wrap up and sustainability planning

Desired outcomes

• Reflection on coaching experience

• Sustainability plan

• Coaching post assessment

Guiding Questions

• What were your greatest learnings from this coaching experience?

• How will you ensure you continue to make progress toward your development goal?

• Who could help you stay accountable to this plan?
