# Supplementary material for: Developing a feedback-rich culture in academic medicine: the effect of coaching and 360-feedback on physician leadership
Source: BMC Med Educ. 2022 Oct 24;22:733. doi: 10.1186/s12909-022-03809-6 (PMC9590387; doi:10.1186/s12909-022-03809-6)
Supplement: Supplementary file 3 — Additional file 3: Appendix C. [file 12909_2022_3809_MOESM3_ESM.docx]

Appendix C: Interview Guide

Thank you for participating in the Leadership Coaching and 360 Degree Assessment program. The main purpose of this interview is to reflect on the sessions this year and gather your feedback on how to make them as valuable as possible. If at any time during the interview you want to stop, we can stop. If at any time after the interview you don’t wish your interview to be included, we can also remove it.

1. How would you describe your experience with the 360 degree coaching program?

2. Prior to starting the coaching program, what were you hoping to accomplish in terms of growing your leadership skills?

3. How did your involvement in the coaching program influence your progress towards those goals?

4. What feedback on the 360 was most valuable to you for your development?

5. How did coaching contribute to the effectiveness of the 360?

6. What kind of feedback did you receive from stakeholders (e.g., your manager, other peers who would be influenced by your progress towards the goals you identified) during the study period? Was there additional feedback that would have been helpful to you?

7. What aspects of your coaching relationship contributed to its effectiveness? [prompts: consistency? rapport? What did they do that helped you learn best?]

8. What aspects of your coaching relationship were challenging? What could have been improved to make it more effective?

9. Would you recommend coaching to others? [why/why not?]

10. What barriers impacted your involvement in the 360 degree coaching program?

11. What factors facilitated your involvement in the 360 degree coaching program? [prompt: Are these factors systemic or individual?]

12. What modifications would you make to the 360 degree coaching program? [Probe: How did the pacing of the coaching sessions feel--would more or less frequent sessions be more useful to you? How many sessions do you think would be most effective?]

13. What additional support do you feel would have further accelerated your progress towards your goals?

14. What insights did you learn about yourself from this process that you can use to be a more effective leader?

15. How do you see this insight changing your leadership style?

16. What barriers to you see in further developing your own leadership effectiveness, and how can the division help?

17. How could we help sustain these changes?

18. Any other comments or questions you wished we’d asked?
